# Is Hardware Removal Recommended after Ankle Fracture Repair?

**DOI:** 10.1155/2016/5250672

**Published:** 2016-10-13

**Authors:** Hong-Geun Jung, Jin-Il Kim, Jae-Yong Park, Jong-Tae Park, Joon-Sang Eom, Dong-Oh Lee

**Affiliations:** ^1^Department of Orthopedic Surgery, Konkuk University School of Medicine, 4-12 Hwayang-dong, Gwangjin-gu, Seoul 143-729, Republic of Korea; ^2^Department of Orthopedic Surgery, Hallym University Sacred Heart Hospital, 896 Pyungchon-dong, Dongan-gu, Anyang-si, Gyeonggi 431-070, Republic of Korea; ^3^Department of Orthopaedic Surgery, Myongji Hospital, 697-24 Hwajung-dong, Deokyang-gu, Goyang-si, Gyeonggi-do 412-270, Republic of Korea

## Abstract

The indications and clinical necessity for routine hardware removal after treating ankle or distal tibia fracture with open reduction and internal fixation are disputed even when hardware-related pain is insignificant. Thus, we determined the clinical effects of routine hardware removal irrespective of the degree of hardware-related pain, especially in the perspective of patients' daily activities. This study was conducted on 80 consecutive cases (78 patients) treated by surgery and hardware removal after bony union. There were 56 ankle and 24 distal tibia fractures. The hardware-related pain, ankle joint stiffness, discomfort on ambulation, and patient satisfaction were evaluated before and at least 6 months after hardware removal. Pain score before hardware removal was 3.4 (range 0 to 6) and decreased to 1.3 (range 0 to 6) after removal. 58 (72.5%) patients experienced improved ankle stiffness and 65 (81.3%) less discomfort while walking on uneven ground and 63 (80.8%) patients were satisfied with hardware removal. These results suggest that routine hardware removal after ankle or distal tibia fracture could ameliorate hardware-related pain and improves daily activities and patient satisfaction even when the hardware-related pain is minimal.

## 1. Introduction

Displaced ankle and distal tibia fractures are among the most common fractures of the extremities and are often treated by open reduction and internal fixation (ORIF) [1, 2]. However, whether hardware should be routinely removed after bony union has been achieved and in the absence of substantial hardware-related pain is controversial [3–5], in part because few studies have addressed this question.

Pain from soft tissue irritation when normal activities resume after fracture healing is typical indication for removing implants from adults [3]. The concerns with retaining metal implants include deep late infection, metal allergy or toxicity, tumorigenicity, hardware migration, metal failure, and secondary fracture at plate ends [6]. However, the recommended indications for hardware removal in surgical textbooks differ. Recently, Hanson et al. reported that many surgeons favor leaving implants* in situ* and are unconvinced of clinically significant adverse effects, but this report described only surgeons' opinions and practice patterns and did not consider the topic from the patient's perspective [3].

The incidence of late pain at fracture hardware sites and rate of hardware removal for late pain for ankle or distal tibia fractures are also not well documented. Moreover, little information is available on the relative merits of hardware removal after ORIF, and almost no studies have considered the change of patients' functional status related to daily activities after routine hardware removal [3, 7]. Therefore, the purpose of this study was to determine the clinical effects in the patients' perspective of routine hardware removal after bony union of ankle or distal tibia fractures, irrespective of the degree of hardware-related pain.

## 2. Patients/Materials and Methods

Institutional Review Board approval was obtained for this study and informed consent was obtained from all patients involved. We had used some implants which included 3.5 mm cortical screws, 4.0 mm partial-threaded cannulated screws, one-third tubular plates, and/or locking compression plates (Synthes AG, Bettlach, Switzerland) for treating all these fractures. This study included patients who already had been initially treated by ORIF at our institution from July 2006 to July 2010 and enrolled consecutive 80 ankle or distal tibial fractures that underwent hardware removal after fracture bony union. Patients with intra-articular distal tibia fractures were excluded because intra-articular joint pain could confound the results.

Hardware removal was routinely recommended only after a noneventful course and radiographically confirmed bony union, usually about 1 year after surgery, even when hardware-related pain or ill-defined ankle discomfort was minimal. Hardware was not removed from the patients with age of more than 65 years considering the life expectancy. Patients were followed up for at least 6 months after removal.

Patients were interviewed at the clinic by one of the authors (JIK) who used a custom-made questionnaire to evaluate the functional changes in daily activities after hardware removal as shown as follows. 

*Patient's Evaluation after Hardware Removal*

*Ankle and Distal Tibia Fractures ORIF*

- Date (year/month/day): ____/____/_____
- Patient's name: _______________________
- Registration number: _______________________
- Sex/Age: ______/________
- Mobile phone number: ___-___-__________
- Home phone number: ___-___-__________
- Occupation: _____________ Daily activity level (high, intermediate, low)
- Type of medical insurance: (Private/Occupational/National)
- Name of diagnosis: ____________________
   Ankle (MM, LM, BM, TM)/Tibia (distal, shaft)/Calcaneus/Talus/Lisfranc
- Name of operation: ____________________
   (Plate & screw, screw, IM nail, K-wire), broken hardware (Y/N)
- Date of operation (year/month/day): ___/____/________
- Date of Internal fixation implant removal (year/month/day): ____/____/_______
   (Post-op:   months)
- Site of pain:
- Level of pain (VAS score) preop:  postop:
   none
    □ 0
   low
    □ 1
    □ 2
   mild
    □ 3
    □ 4
   intermediate
    □ 5
    □ 6
    □ 7
   severe
    □ 8
    □ 9
   extremely severe
    □ 10
- Level of pain on a wet and gloomy weather
  - extremely painful
  - more painful
  - no difference from other days
  - reduced
  - very reduced
- How is your postoperative ankle joint movement?
  - much more comfortable
  - more comfortable
  - no change
  - uncomfortable
  - much more uncomfortable
- What's your level of satisfaction about operation scar?
  - very satisfied
  - satisfied
  - fair
  - unsatisfied
  - very unsatisfied

(6) How is swelling on operation lesion compared to pre-implant removal state?
  - very reduced
  - reduced
  - no change
  - increased
  - very increased
(7) How is skin sensation on operation lesion compared to pre-implant removal state?
  - very sensitive
  - sensitive
  - no change
  - dull
  - very dull
(8) Do you have any tingling sense on either operation lesion or other body parts after the implant removal?
   
*Please specify the body parts.*
     ——(Yes/No)
     ——(Yes/No)
     ——(Yes/No)
(9) Function evaluation
  (9.1) How was the “level of limping” accommodated after the surgery?
    (a) much improved
    (b) improved
    (c) no change
    (d) more limping after the surgery
    (e) much more limping after the surgery
  (9.2) How was the “level of discomfort when walking up the stair” accommodated after the surgery?
    (a) very much improved
    (b) improved
    (c) no change
    (d) more uncomfortable after the surgery
    (e) more uncomfortable after the surgery
  (9.3) How was the “level of discomfort when squatting” accommodated after the surgery?
    (a) very much improved
    (b) improved
    (c) no change
    (d) more uncomfortable after the surgery
    (e) much more uncomfortable after the surgery
  (9.4) How was the “level of discomfort at work” accommodated after the surgery?
    (a) very much improved
    (b) improved
    (c) no change
    (d) more uncomfortable after the surgery
    (e) much more uncomfortable after the surgery
  (9.5) How was the “level of discomfort during sports activities” accommodated after the surgery?
    (a) very much improved
    (b) improved
    (c) no change
    (d) more uncomfortable after the surgery
    (e) much more uncomfortable after the surgery

(10) What is your general level of satisfaction about hardware removal surgery?
  - very satisfied
  - satisfied
  - fair
  - unsatisfied
  - very unsatisfied
(11) If you were in the same situation, would you receive the same surgery again?
  - Yes, I'd like to.
  - No, I wouldn't.
  - not determined.
(12) Would you recommend the hardware removal surgery to others?
  - Yes, I'd like to.
  - No, I wouldn't.
  - not determined.
(13) Please specify your recommendation if you have.
  - AOFAS score: Pre-hardware removal:
  - Post-hardware removal:

All interviews were performed by one of the authors (JIK). Pain was assessed on a 10 cm visual analog scale (VAS) anchored at one end by “no pain” and the other as “worst pain imaginable.” Other questions evaluated ankle stiffness and change in ankle discomfort while walking on an uneven surface, while walking upstairs, and while squatting. In addition, information was collected on patient satisfaction, intermittent swelling, and surgical scar formation.

### 2.1. Statistical Analysis

When comparing patients of tibia fracture with patients of ankle fracture, we used VAS pain score as the primary outcome indicator and determined effect size as 1 for retrospective power analysis. Thus a power analysis indicated that a sample size of 24 would provide 90% statistical power to detect an effect of this size between the two groups (*α* = .05, *β* = .1) with use of Mann–Whitney test. Therefore, comparing two groups (*N* = 24, 56) met the statistical power requirements of this study. In comparing preoperative and postoperative pain, Wilcoxon signed ranks test was used. Fisher's exact test was used for comparing the frequency of complications. We used Spearman correlation test to analyze associations between patient's satisfaction and other independent factors: age, preoperative and postoperative pain scores, scar improvement, swelling improvement, and screw breakage.

Logistic regression analysis was also performed with variables significant at the 0.1 level in univariable analysis. To avoid the idea that this study appeared more significant than it actually is, we analyzed mildly symptomatic patients (pain score ≤ 3) separately. In the last 33 cases enrolled, before hardware removal we asked patients whether they would undergo hardware removal if clinician did not recommend surgery. Following that, responses were compared between patients who would and would not undergo hardware removal without a clinician's recommendation.

Unless otherwise noted, the significance level for all statistical tests was set at 5% and all tests were two-tailed. Statistical analysis was performed using SPSS software (version 20, IBM Inc., New York) which was utilized throughout for this statistical analysis.

## 3. Results

We studied 80 fractures in 56 men and 22 women, all of whom had been followed up for more than 6 months after hardware removal (mean, 17; range 7 to 37 months). Mean age at hardware removal was 41.8 (range, 18 to 64) years. The 56 ankle fractures were classified using the Lauge-Hansen criteria: 39 (69.6%) of the 56 were supination-external rotation type, 3 (5.4%) were supination-adduction type, 13 (23.2%) were pronation-external rotation type, and 1 (1.8%) was pronation-abduction type. All 24 distal tibia fractures were classified as 43A according to the OA/Orthopaedic Trauma Association Classification [8, 9]. A plate and screws were used for internal fixation in 64 patients (66 fractures) and only screws were used in 14 patients (14 fractures; Table 1).

Radiographic bony union was confirmed for all 78 patients with 80 fractures, and all were followed up long enough after ORIF to have reached maximal recovery before scheduled hardware removal. When the achievement of bony union was uncertain on simple radiograph and physical examination, the confirmation of union was obtained by computerized tomography (CT). Consequently, the mean time from index fracture surgery to hardware removal was 23.6 (range 7 to 240) months.

Mean pain scores were significantly lower after hardware removal overall and for ankle and tibial fractures, both after fixation with plates or screws, and among patients with preremoval pain scores of 3 or less (Table 2).

Regarding hardware types, mean VAS pain score in the plate fixation group (66 fractures) decreased significantly from 3.4 (range 0 to 6) to 1.3 (range 0 to 6) (*p* < .001). Similarly, mean VAS score in the screw fixation group (14 fractures) significantly decreased from 3.2 (range 0 to 5) before to 1.1 (range 0 to 3) after hardware removal (*p* < .001). Again, no significant difference was found between these two groups (*p* = .868) (Table 2).

About 72% of all patients reported subjective improvements in ankle stiffness, while about 89% of the patients improved walking on uneven ground, climbing stairs, and squatting (Table 3). 63 (80.8%) patients were satisfied with hardware removal and only three patients were dissatisfied with hardware removal, one of whom initially complained of ankle pain from posttraumatic ankle arthritis. Among all 78 patients, 73 (94%) said they would be willing to undergo hardware removal surgery again under similar circumstances, and 69 (88%) said they would recommend removal surgery to others. Satisfaction was affected by postoperative pain VAS, scar improvement, swelling improvement, and screw breakage (Table 4).

Complications of screw breakage occurred in four patients, two of whom were with distal tibia fractures and the others with ankle fracture. None complained of symptoms because of remaining screw fragment. The frequency of that complication did not differ significantly between the two groups (*p* = .347). The degree and severity of intermittent ankle swelling decreased in 58 (72.5%) patients, while it increased in 4 (5%).

In subgroup analysis, all 43 patients who had minimal preoperative pain (scores ≤ 3) significantly improved from the mean score of 2.21 to the mean of 0.77 (Table 2). Of this same patient group, 37 (86%) were satisfied with hardware removal. Interestingly, the satisfaction of patients with even lower preoperative pain scores (VAS ≤ 3) was more affected by postoperative pain score than any other factors, such as scar or screw breakage (Table 5).

Of the last 33 patients enrolled, 24 (73%) underwent hardware removal on the clinician's recommendation rather than their own request, and 18 (75%) of the 24 patients reported satisfaction with hardware removal surgery. Another five patients all showed fair response although their pain was reduced. Only 1 (4.17%) of the 24 patients was dissatisfied with the surgery, the reason given being a hypertrophic scar and broken screw.

## 4. Discussion

ORIF of displaced ankle fractures is one of the most common surgeries in the orthopedic field and had showed better outcomes than closed reduction and cast immobilization [1, 10, 11]. However, internally fixed hardware can cause pain, limit ankle motion, and result in skin protrusions or discomfort because of muscle deficiencies in the region and the anatomical proximities of skin and bones [4, 7]. Jacobsen et al. reported that 89.4% of internally fixed ankle fractures result in reports of discomfort, such as soreness over implants and scar tissue, reduced ankle joint movement, and strain-related pain [4]. The removal of fracture fixation hardware was found to be comprised of 30% of planned and 15% of total surgeries in a Finnish study and of 5% of all orthopedic surgeries in a study conducted in the USA [6, 12].

However, the indications and the clinical need for routine hardware removal after the treatment of distal tibia or ankle fractures by ORIF are disputed, especially when hardware-related pain is negligible [13]. Thus, the decision is usually made by surgeon's preference [3]. Some physicians recommend removal for all patients, whereas others recommend it for only young, active patients, because hardware removal has been reported to pose potential risks of neurovascular injury, refracture, and implant breakage [4, 6]. Accordingly, the benefits of the procedure had to outweigh these risks, although, anecdotally, daily activities appear to improve in most of these patients after hardware removal and most appear to be satisfied with the removal procedure [3, 7, 13]. Through this study, we tried to verify that hardware removal in these patients with mild hardware-related pain improves the functional status in patient's perspective and patient satisfaction.

Previous outcome studies on hardware removal have been limited to hardware-related pain. Brown et al. [7] and Keating et al. [14] found complete relief from knee pain after hardware removal in 45% and 27% of all cases, respectively, after tibia nail removal, whereas Jacobsen et al. [4] and the present study found that 75% and 80.77%, respectively, of patients with an ankle fracture were satisfied with hardware removal [4, 14, 15]. These high satisfaction rates can be partly explained by the superficial locations of hardware in the ankle and distal tibia area [4, 7]. Williams et al. recently reported the benefits of implant removal from the foot and ankle only in cases of hardware-related pain [13]. But, we routinely performed implant removal to all the patients regardless of their pain degree and surveyed other symptoms.

Our study has several limitations that warrant consideration. First, we did not use a control group. Determining the effect of routine implant removal by a randomized trial, such as sham procedure, is difficult for ethical or methodological reasons. For example, patients can easily know the existence of their implant because hardware around ankle joint is usually prominent onto skin, which prevents blinded study and placebo effect. Therefore, we recommended routinely removing hardware from all patients and separately analyzed subgroups that had mild symptom and therefore less motivation to have hardware removed. Second, standard functional evaluation score, such as American Orthopaedic Foot and Ankle Society ankle-hindfoot functional score, was not performed. But this was because the patients did not complain of significant pain or other discomfort expecting functional impairment. Findings of physical examination such as ankle movement also were not included in questionnaire of this study, which could have been less biased by the patients' expectations. Despite these limitations, we believe that this outcome of study could be used to develop prospective cohort or randomized controlled trials focusing on genuine effects of routine hardware removal.

## 5. Conclusions

Our results of hardware removal after ORIF for an ankle or distal tibia fracture, even in mild symptoms, improve the functional daily activities in the patients' perspective and high patient satisfaction, supporting the recommendation to routinely remove hardware from these patients.

## Figures and Tables

**Table 1:** Characteristics of patients with fractures of the ankle or distal tibia repaired with open reduction and internal fixation in a study of routine hardware removal (*n* = 80 feet with 78 patients).

Demographic	Count
Age (years)^*∗*^	41.8 ± 15.5
Sex	
Male	47 (58.8%)
Female	33 (41.3%)
Injury side	
Right	31 (38.8%)
Left	49 (61.3%)
Hardware	
Plate and screws	67 (83.8%)
Screws only	13 (16.3%)
Location	
Ankle	56 (70.0%)
Distal tibia	24 (30.0%)
Time interval between two surgeries (months)^†^	23.6 (range 7 to 240)

^*∗*^Mean ± SD.

^†^Mean (range).

**Table 2:** Pain scores before and after hardware removal from 78 patients with fractures of the ankle or distal tibia (*n* = 80 feet).

Characteristic	Pain score^*∗*^		*p* value
	Preoperative	Postoperative	
*Location*			.500^†^
Ankle (*n* = 56)	3.5 ± 1.6	1.3 ± 1.0	<.001
Distal tibia (*n* = 24)	3.4 ± 1.6	1.2 ± 1.2	<.001
*Hardware type*			.614^†^
Plate and screws (*n* = 67)	3.4 ± 1.6	1.3 ± 1.2	<.001
Screws only (*n* = 13)	3.2 ± 1.7	1.1 ± 1.0	<.001
*Severity of pain*			
VAS ≤ 3 (*n* = 43)	2.2 ± 1.0	0.8 ± 0.8	<.001

*Overall (n* = 78)	3.4 ± 1.6	1.3 ± 1.2	<.001

^*∗*^Mean ± SD.

^†^Difference of postoperative pain VAS between two groups.

**Table 3:** Changes in ankle function reported by 78 patients with fractures of the ankle or distal tibia (*n* = 80) after hardware removal.

Outcome	Change in function after hardware removal		
	Better	No change	Worse
	*n* (%)	*n* (%)	*n* (%)
Ankle stiffness	58 (72.5)	14 (17.5)	8 (10.0)
Walking on uneven ground	66 (82.5)	12 (15.0)	2 (2.5)
Discomfort on walking up the stairs	65 (81.3)	13 (16.3)	2 (2.5)
Discomfort when squatting	64 (80.0)	11 (13.8)	5 (6.3)
Discomfort at physical exercise (e.g., jogging, basketball, and hiking)	61 (76.3)	12 (15.0)	7 (8.8)

**Table 4:** Factors associated with patient satisfaction in 78 patients with fractures of the ankle or distal tibia (*n* = 80) after hardware removal.

Variables	Coding	Univariable analysis		Logistic regression analysis	
		*R*	*p* value	Odds ratio	*p* value
Age	Years	.15	.20		
Gender	M/F (0/1)	−.12	.30		
Preoperative pain^†^	0 to 10	−.09	.44		
Postoperative pain^†^	0 to 10	−.32	.004^*∗*^	.52	.06
Improved scar	Grade (0~2)	.39	<.001^*∗*^	17.2	.01
Improved swelling	Grade (0~2)	.37	.001^*∗*^	13.4	.07
Screw breakage	No/yes (0/1)	−.33	.003^*∗*^	.03	.03

^*∗*^Indicating significance.

^†^10 cm visual analog scale.

**Table 5:** Factors associated with patient satisfaction in 43 patients, 43 feet with mild pain group.

Variables	Coding	Pain score ≤ 3 (*n* = 43)	
		*R*	*p* value
Age	Years	0.02	.92
Gender	M/F (0/1)	−0.03	.86
Preoperative pain^†^	0 to 10	0.16	.30
Postoperative pain^†^	0 to 10	−0.36	.02^*∗*^
Improved scar	Grade (0 to 2)	0.17	.28
Improved swelling	Grade (0 to 2)	0.39	.01^*∗*^
Screw breakage	No/yes (0/1)	−0.23	.14

^*∗*^Indicating significance.

^†^10 cm visual analog scale.

## References

[1] Brodie I. A., Denham R. A. (1974). The treatment of unstable ankle fractures. *The Journal of Bone & Joint Surgery—British Volume*.

[2] Donatto K. C. (2001). Ankle fractures and syndesmosis injuries. *Orthopedic Clinics of North America*.

[3] Hanson B., van der Werken C., Stengel D. (2008). Surgeons' beliefs and perceptions about removal of orthopaedic implants. *BMC Musculoskeletal Disorders*.

[4] Jacobsen S., de Lichtenberg M. H., Jensen C. M., Tørholm C. (1994). Removal of internal fixation—the effect on patients' complaints: a study of 66 cases of removal of internal fixation after malleolar fractures. *Foot and Ankle International*.

[5] Loder R. T., Feinberg J. R. (2006). Orthopaedic implants in children: survey results regarding routine removal by the pediatric and nonpediatric specialists. *Journal of Pediatric Orthopaedics*.

[6] Böstman O., Pihlajamäki H. (1996). Routine implant removal after fracture surgery: a potentially reducible consumer of hospital resources in trauma units. *Journal of Trauma—Injury, Infection and Critical Care*.

[7] Brown O. L., Dirschl D. R., Obremskey W. T. (2001). Incidence of hardware-related pain and its effect on functional outcomes after open reduction and internal fixation of ankle fractures. *Journal of Orthopaedic Trauma*.

[8] Lauge-Hansen N. (1950). Fractures of the ankle. II. Combined experimental-surgical and experimental-roentgenologic investigations. *Archives of Surgery*.

[9] (1996). Fracture and dislocation compendium. Orthopaedic Trauma Association Committee for Coding and Classification. *Journal of Orthopaedic Trauma*.

[10] Mast J. W., Teipner W. A. (1980). A reproducible approach to the internal fixation of adult ankle fractures: rationale, technique, and early results. *Orthopedic Clinics of North America*.

[11] Mitchell W. G., Shaftan G. W., Sclafani S. J. A. (1979). Mandatory open reduction: its role in displaced ankle fractures. *Journal of Trauma—Injury, Infection and Critical Care*.

[12] Rutkow I. M. (1986). Orthopaedic operations in the United States, 1979 through 1983. *The Journal of Bone & Joint Surgery—American Volume*.

[13] Williams A. A., Witten D. M., Duester R., Chou L. B. (2012). The benefits of implant removal from the foot and ankle. *Journal of Bone and Joint Surgery—Series A*.

[14] Keating J. F., Orfaly R., O'Brien P. J. (1997). Knee pain after tibial nailing. *Journal of Orthopaedic Trauma*.

[15] Court-Brown C. M., Gustilo T., Shaw A. D. (1997). Knee pain after intramedullary tibial nailing: its incidence, etiology, and outcome. *Journal of Orthopaedic Trauma*.

